# Supplementary material for: Are carbon emissions trading and green financial instruments synergistic? -Comprehensive quantitative research based on content analysis
Source: PLoS One. 2024 Mar 7;19(3):e0298601. doi: 10.1371/journal.pone.0298601 (PMC10919601; doi:10.1371/journal.pone.0298601)
Supplement: S1 Data — (ZIP) [file pone.0298601.s001.zip › Data/地级市Z_F工作报告.zip!/╣≤╓▌/╣≤╤⌠╩╨/└┤╫╘╣1⁄2╓┌║┼í╛┬φ┐╦╩2╛▌═°í┐_2019.docx]

各位代表：

　　现在，我代表市人民政府向大会报告工作，请予审议，并请市政协委员和其他列席人员提出意见。

　　一、2018年政府工作回顾

　　时节如流，日新月异。刚刚过去的2018年，面对严峻复杂的国际形势和繁重艰巨的改革发展稳定任务，我们以习近平新时代中国特色社会主义思想为指导，深入学习贯彻党的十九大精神、习近平总书记对贵州工作系列重要指示批示精神，在中共贵阳市委的坚强领导下，牢记嘱托、感恩奋进，统筹推进“五位一体”总体布局、协调推进“四个全面”战略布局，以践行新发展理念引领高质量发展。牢牢守好发展和生态两条底线，坚决打好防范化解重大风险、精准脱贫、污染防治三大攻坚战，纵深推进大扶贫、大数据、大生态三大战略行动，以新作为带来新气象，以新斗争开启新局面，经济社会发展取得了可观可感的实绩，建设公平共享创新型中心城市收获了令人鼓舞的成果。

　　经济发展态势良好。地区生产总值完成3798.45亿元，增长9.9%；社会消费品零售总额达到1299.47亿元，增长8%；固定资产投资增长15%。经济增速有望连续6年位居全国省会城市第一，经济总量持续增大，全面小康指数在全国294个地级城市中位列第49名，荣获“影响中国”2018年度城市称号。

　　新旧动能加速转换。新经济增加值占地区生产总值比重达到21%，比上年提高2个百分点，大数据与实体经济融合指数达到45.3，全社会研究与试验发展经费投入占地区生产总值比重提高到1.7%，科技进步对经济增长的贡献率达到61%，分别比上年提高0.36和0.67个百分点。

　　质量效益不断提高。绿色经济增加值占地区生产总值比重达到42%，比上年提高3个百分点；规模以上工业企业利润总额增长15.8%，比上年提高14.3个百分点；完成一般公共预算收入411.34亿元，增长8.9%，一般公共预算支出627.54亿元，增长7.7%；城乡居民人均可支配收入分别达到35115元、15648元，分别增长9.1%、9.7%，与经济增长基本保持同步。

　　改革开放成果丰硕。全市日均新增市场主体286户，市场主体总量达到49.4万户，在2018年中国大陆最佳商业城市100强中排名第27位，比2017年上升38位，荣获2018年中国国际营商环境标杆城市称号，开通洲际航线3条，填补了贵州没有洲际航线的空白。

　　环境保护力度加大。集中式饮用水源地水质100%稳定达标，主要河流国控、省控断面水质优良率达到93.75%；PM2.5等六项污染物浓度均低于国家二级标准，空气质量优良率达到97.8%，比2017年提高2.7个百分点，在全国169个大气污染防治重点城市中列第11位；森林覆盖率达到52%，比2017年提高3.34个百分点。

　　民生福祉持续增进。城镇登记失业率3.1%;居民消费价格上涨1.7%，物价水平保持平稳，惠民生鲜超市总量达到154个，实现行政社区全覆盖;学前三年毛入园率达到93.05%，九年义务教育巩固率达到95.03%，高中阶段教育毛入学率达到93.52%；社区卫生服务中心标准化建设达标率超过80%，公立医院药占比降至30%以下，食品药品抽检合格率分别达到98.94%、99.5%，创历史新高。

　　一年来，我们付出很多，也收获满满。全市人民挥洒的汗水已经成为高质量发展的累累硕果，正在转化为推动贵阳经济社会加快发展、跨越发展的强大动力。

　　（一）尽锐出战、精准发力，三大战役战果丰硕。防范化解重大风险攻坚战有力有效。进一步统筹政府债务管理，依法依规开展融资工作。实施PPP项目17个，总投资755.3亿元，整治非法金融机构27家，取消12家融资担保机构、20家小额贷款公司经营资格，牢牢守住了不发生区域性系统性金融风险的底线。精准脱贫攻坚战强力推进。脱贫攻坚“春风行动”“夏秋攻势”“秋后算账”“冬季充电”连战连捷，“五个专项治理”深入推进。1462名非兜底保障贫困人口出列，建成“组组通”公路5294.4公里，30户以上村民组全部通硬化路，易地扶贫搬迁工程入住519户、2042人，建档立卡贫困人口全部纳入健康扶贫对象，教育精准扶贫实现贫困家庭全覆盖，惠及贫困学生1.66万人；大力推动贵阳大市场带动全省大扶贫，实施对口帮扶项目59个，安排对口帮扶资金1.49亿元，采购本省农产品120万吨，提供就业岗位17.6万个，带动省内10万贫困人口稳定脱贫。污染防治攻坚战成效明显。深入开展中央环保督察反馈问题和“回头看”发现问题整改，群众反映的环保突出问题全面整改。中央环保督察反馈的32个问题整改基本完成，1681件群众信访投诉件全部办结，群众满意度达到86%；建设再生水厂18个，投入运营9个，日新增污水处理能力20.08万吨，红枫湖饮用水源地一级保护区内房屋全部搬迁，19条排污大沟整治完成，七彩湖等33个黑臭水体得到有效治理，花溪城市生活垃圾综合处理项目运营；全年查处环境违法案件237起、林业违法案件641起；通过全国水生态文明城市建设试点国家验收，南明河实现除臭变清目标。爽爽的贵阳天更蓝、山更绿、水更清，老百姓生活更舒畅。

　　（二）结构优化、产业壮大，实体经济加快发展。供给侧结构性改革扎实推进。“三去一降一补”工作深入开展，全年淘汰落后产能99万吨，减免税费203.96亿元，完成补短板投资956.76亿元，三次产业结构调整为4:37.2:58.8；质量强市战略深入推进，获得第五届中国工业大奖项目表彰奖1个、省长质量奖2个、省长质量奖提名奖2个，颁发第二届市长质量奖2个、市长质量奖提名奖2个。农村产业革命成效显著。农业产业结构调整完成82.78万亩，种植特色农产品200万亩，乡村旅游收入150亿元，新增省级农业高效示范园区4个，新认证“三品一标”农产品161个，成功举办贵州省首届中国农民丰收节等系列活动，一、二、三产业加快融合，一产增加值完成153.1亿元，增长6.6%，现代农业增加值占一产增加值比重提高到39%。工业企业加速转型。“双千工程”“万企融合”行动强力推进，全年新引进工业项目276个，新增规模以上工业企业92个，规模以上工业增加值增长7.4%，“千企改造、一企一策”完成投资175亿元，4个项目成为国家2018年智能制造试点示范项目，贵州工业互联网平台成为国家2018年工业互联网创新发展工程，吉利汽车批量生产，吉利汽车发动机、汉能汉瓦产品顺利下线，上云工业企业超过500个。现代服务业提档升级。现代服务业增加值占服务业增加值比重提高到53%，观山湖现代服务产业试验区成立；金融机构人民币存贷款余额分别达到11357.44亿元和12412.75亿元，增长5%和19.3%，中国进出口银行贵州分行营业，浙商银行贵阳分行筹建；接待游客1.88亿人次，增长26.7%，实现旅游总收入2456.56亿元，增长31.2%，入境游客54万人次，增长32%，新增4A级旅游景区3个，荣获2018年度全国最佳优质旅游城市，连续四年荣膺全国最佳避暑旅游城市，国家全域旅游示范区建设加快推进；建成贵阳农产品物流园、普洛斯配送中心等大型物流基地，成为国家物流枢纽承载城市。

　　（三）创新驱动、深度融合，数字经济蓬勃发展。大数据产业不断壮大。大数据企业主营业务收入达到1000亿元，增长22.4%；互联网出省带宽达到9130Gbps，通信光缆超过19万公里，行政村100%通光纤，市域4G网络全覆盖，成为全国首批5G应用示范城市，入选“宽带中国”优秀城市；服务器总量达到28.5万台，中电西南云计算中心等3个数据中心成为国家绿色数据中心试点单位；建成大数据产业园10个，引进大数据优强企业58个，新增规模以上大数据企业51个，满帮集团成为福布斯中国50家最具创新力企业，易鲸捷荣获2018世界物联网博览会银奖，贵阳大数据交易所可交易数据量超过150PB。大数据应用不断拓展。政府数据实现100%共享交换，政府数据开放水平全国第一，政务系统100%接入“云上贵州”平台，互联网+政务服务平台成为2018年度全国政府网站十大优秀创新案例；推出中国数谷大数据应用场景100个，培育典型应用示范项目44个，实施大数据融合标杆项目36个、融合示范项目300个，中国人工智能开放创新平台等大数据应用创新平台加快建设，贵阳大数据创新产业（技术）发展中心成为国家级众创空间。大数据法规不断完善。《贵阳市大数据安全管理条例》《贵阳市政府数据资源管理办法》《贵阳市政府数据共享开放实施办法》《贵阳市政府数据共享开放考核暂行办法》等法规规章施行。大数据标准不断充实。参与交易数据描述、数据质量评价指标等国家标准制定，人口基础数据、法人单位基础数据等地方标准施行，国家技术标准创新基地（贵州大数据）贵阳区域分基地成立。大数据安全不断加强。政府网络安全指数在全国36个中心城市中排名第10位，大数据安全产业示范区（一期）、国家大数据安全靶场（一期）、态势感知综合防护平台（一期）建成，大数据及网络安全技术创新中心、应用示范中心成立，贵阳大数据及网络安全攻防演练成功举行，贵阳经济技术开发区成为全国首个大数据安全认证示范区，全市网络安全防护能力大幅提升。创新的贵阳正在驶入大数据发展快车道，中国数谷的影响力和美誉度越来越大。

　　（四）交通引领、建设发力，城乡面貌日新月异。交通基础设施加快完善。新增普通国省干线公路82公里、乡镇连接高速公路联络线42公里，创建“四好”农村路600公里，提等改造农村公路103公里，高速公路通车里程602公里，花冠路、同城大道、北京东路延伸段等一批重大路网项目建成通车，人民大道北段基本建成，新东二环提质完成；渝贵高铁开通，贵南高铁加快建设，高铁贵阳东站全面运营，十字型高铁网初步形成；贵阳龙洞堡国际机场3号航站楼开工建设、三期扩建工程顺利推进。人居环境质量加快改善。实施棚户区城中村改造960.08万平方米，惠及21.15万人，改造老旧小区院落158个，整治背街小巷200公里；农村危房改造完成9376户，农村老旧住房整治3404户，小康房建设2700户，建设田园综合体30个，建成富美乡村示范村30个；新建和提升改造城区公厕203座、农村公厕376座、农村户用卫生厕所1.25万户。城乡融合水平加快提升。常住人口城镇化率达到75.4%；镇（乡）村建筑风貌导则、行政村村域规划以及30户以上农村居民点建设规划编制完成；新建和改造供水管网118公里，建成中小型水库5座，“小康水”项目完成194个，17.7万人饮水安全和6.5万亩耕地灌溉问题得到有效解决；新增和改造110千伏及以下农村电网4481.06公里、配电变压器788台，农村供电可靠率达到99.9%，建制村通邮率达到99.4%，快递网点乡镇全覆盖，城市基础设施、公共服务设施正在加快向农村延伸、向乡镇覆盖。

　　（五）深化改革、锐意进取，活力动能持续增强。营商环境持续优化。“放管服”改革深入推进，取消各类证明材料757项，企业和群众办事需提供的材料减少30%以上，一次办成的政务服务事项达到48个，企业开办时间缩减至5个工作日以内，政务服务“一网通办”作为典型经验受到国务院办公厅通报表扬；成为全国首批工程建设项目审批制度改革试点城市，工程建设项目审批时限压减至100天以内；营商环境指数排名从2017年的第30位跃升至第14位，企业和群众办事更方便、更快捷、更高效、更舒心。重点领域改革持续深入。市级和区（市、县）机构改革工作有序推进，国有企业战略性重组进展顺利，公立医院综合改革全面推开，生态文明体制机制改革不断完善；实施城市“三变”改革项目40个，惠及城市居民23.2万人，完成300个行政村农村集体产权制度改革、642个农村“三变”改革试点工作，24.28万农民变成股东。改革的贵阳全面发力，改出了前进的新动力，改出了发展的新天地。

　　（六）增进交流、深化融合，开放大门越开越大。开放通道加速拓展。开通贵阳至旧金山、洛杉矶、莫斯科洲际航线，贵阳龙洞堡国际机场旅客吞吐量突破2000万人次，高铁直达香港。开放平台不断做优。中国（贵阳）跨境电子商务综合试验区设立；2018中国国际大数据产业博览会、生态文明贵阳国际论坛2018年年会等重大活动成功圆满举办；贵阳国家高新技术产业开发区成为国家国际科技合作基地、国家双创示范基地，贵阳经济技术开发区成为国家循环化改造示范试点园区，贵阳综合保税区跨境股权投资国家试点工作取得突破，贵州双龙航空港经济区发展规划实施。开放合作持续深化。东盟—贵州（贵阳）旅游文化推广中心成立，中印IT产业集聚区启动建设，瑞士（贵州）产业示范园加快建设，国际友好交往城市达到17个。开放红利加快释放。进出口总额达到34.94亿美元，增长15.2%，实际利用外资15.89亿美元，增长18.1%；新引进国内外500强企业15家，累计达到113家，新引进项目实际到位资金2218.67亿元，增长35.4%，新增对外投资企业15家、外贸企业74家、外商投资企业54家，贵州轮胎股份有限公司越南项目进展顺利。开放的贵阳通向世界的道路越来越宽，迈向世界的步伐越来越快。

　　（七）和谐共享、普惠于民，人民生活更加幸福。就业更加稳定。全员培训农村贫困劳动力17562人次，发放创业担保贷款2.72亿元，新增城镇就业23.31万人，农村劳动力转移3.82万人，803个建设项目实行农民工实名制管理，充分就业社区（村）创建率达到88%。教育更加均衡。“百校攻坚”行动计划和“全面改薄”工作扎实推进，新建、改扩建中小学、幼儿园276所，新增学位30480个，清理无证校外培训机构2360所，在全省率先开展“乐童计划”，学生托管率达到85%以上。医疗更加普惠。“百院攻坚”行动计划深入推进，新建、改扩建医院13所，建成健康细胞工程示范单位30个，贵阳市人口健康信息云平台运营，全面实现跨省异地住院即时结算。出行更加便捷。轨道交通1号线全线运营、2号线建设加快、3号线开工建设，贵阳进入了轨道交通时代；贵阳牌照小型客车环城高速免费通行，交通互联互通卡使用率达到70.6%，建成人行过街设施30个，修补提质人行道4.7万平方米、车行道8.5万平方米，新增公共停车位1万个，治理堵点30个。 文化更加繁荣。建成基层综合文化服务中心142个、社区图书共享系统15个、全民健身路径工程40个，第十四届亚洲越野跑锦标赛、贵阳市第十三届运动会暨市第十届少数民族传统体育运动会、第六届国际阳明文化节等重大文体活动成功举办。养老更加安心。成为全国第三批中央财政支持开展居家和社区养老服务改革试点，建成城乡社区日间照料中心25个、农村互助幸福院30个。社会更加和谐。通过国家卫生城市复审；全年未发生重大及以上生产安全事故，安全生产实现双降；有效化解矛盾纠纷和信访案件7610件，“两严一降”、扫黑除恶专项斗争、禁毒人民战争战果累累，全市刑事案件发案数下降2.02%、破案数上升3.53%，人民群众安全感指数达到98.98%，成为全国新的社会阶层人士统战工作实践创新推广城市，清镇市成为全国新时代文明实践中心建设试点。国家安全、国防动员、民族宗教、人防、档案、侨务、气象、地方志、双拥优抚、工青妇、红十字、老龄和残疾人等事业全面发展。和谐的贵阳人民群众获得感、幸福感、安全感和对发展的满意度持续上升。

　　（八）忠诚担当、依法守纪，从严治政坚决有力。深入贯彻全面从严治党要求。把政治纪律和政治规矩挺在前面，扎实抓好中央巡视、国务院大督查和省委巡视反馈问题整改，不断加强政府系统党风廉政建设，反腐败斗争取得压倒性胜利并巩固发展。驰而不息兴“三风”、纠“四风”、转作风。坚决贯彻落实中央八项规定及其实施细则精神，认真开展违反“禁酒令”、违规发放津补贴、违规公务接待等突出问题集中专项整治，以过硬的作风解决历史遗留问题463个，化解率达到93.72%，政府执行力和公信力不断提高。纵深推进法治政府建设。修订《贵阳市人民政府工作规则》，开展市政府开放日活动，提请市人大常委会审议地方性法规草案5件，制定政府规章12件，全面清理政府规章200件，办理各级人大代表建议244件、各级政协委员提案358件，办复率均达到100%。

　　各位代表，春华秋实何寻常，如椽巨笔著华章。成就来自牢记嘱托，发展源于感恩奋进。这些成绩的取得，根本就在于以习近平同志为核心的党中央掌舵领航、举旗定向，就在于习近平新时代中国特色社会主义思想的科学指引。这些成绩的取得，是省委、省政府和市委坚强领导、科学决策的结果，是市人大、市政协监督支持、群策群力的结果，更是全市各族人民齐心协力、拼搏奋斗的结果。我代表市人民政府，向全市各族人民致以崇高的敬意！向人大代表、政协委员，向各民主党派、各人民团体，向驻筑人民解放军、武警官兵和人民警察，向中央和省驻筑单位，向贵阳市的老领导、老同志以及所有关心支持贵阳发展的各界人士表示衷心的感谢！

　　凡是过往，皆为序章。成绩属于历史，我们完全有理由为之自豪，但没有理由为之自满，“行百里者半九十”，我们仍需跋山涉水、砥砺前行。我们清醒地认识到，面对中美经贸摩擦带来的不确定性和稳中有变、变中有忧的严峻复杂形势，推动高质量发展仍面临许多困难和挑战，主要是：实体经济不强。工业企业数量不多、体量不大、质量不高，工业化进程滞后于城镇化进程，贵阳作为全省经济社会发展的“火车头”、“发动机”动力不强、马力不足。城市体量不大。城市建设力度不够，城市功能配套不强，产业吸引力、人口集聚力、发展承载力不大，贵阳作为大城市的能级量级不够。开放水平不高。开放型经济规模小，外向型企业数量少，对外合作交流不多，国际化程度不高，贵阳作为国家内陆开放型经济试验区核心区作用发挥不全。创新动力不足。创新体系不健全，创新意识不强烈，创新企业不够多，创新人才不充足，贵阳作为创新型中心城市的创新能力不强。风险隐患不少。随着经济下行压力加大，一些深层次问题和矛盾集中暴露，金融风险、环境风险、安全风险、社会风险不断增多，贵阳作为全省首善之区维护社会和谐稳定的压力不小。担当有为不够。有些地方和部门政策理解力、决策执行力、行动落实力不强，少数干部观大局、谋大事的能力不足，克难攻坚、创新突破的本领不够，贵阳作为省会城市不作为、慢作为、乱作为的现象不少。对这些问题我们要高度重视，认真研究，切实解决，把政府工作做得更好，让省委、省政府和市委放心，让全市人民满意。

　　二、2019年政府工作安排

　　今年是新中国成立70周年，是全面建成小康社会、实现第一个百年奋斗目标的关键之年，是全省脱贫攻坚决战之年，做好政府工作，意义十分重大。

　　当前，世界正面临百年未有之大变局，中国特色社会主义进入了新时代。面对复杂严峻的国际形势，面对繁重艰巨的改革发展稳定任务，我们必须增强“四个意识”，坚定“四个自信”，做到“两个维护”，坚决贯彻落实以习近平同志为核心的党中央决策部署，在省委、省政府和市委的坚强领导下，大力弘扬新时代贵州精神，准确识变，科学应变，守住“稳”的大局，坚定“进”的决心，把大力实施高水平对外开放、加快推动经济高质量发展的宏伟蓝图一笔一笔地绘就在贵阳大地上。

　　今年政府工作的总体要求是：以习近平新时代中国特色社会主义思想为指导，全面贯彻党的十九大和十九届二中、三中全会精神，认真落实中央和全省经济工作会议、市委十届五次、六次全会精神，深入贯彻落实习近平总书记对贵州工作系列重要指示批示精神，牢记嘱托、感恩奋进，统筹推进“五位一体”总体布局，协调推进“四个全面”战略布局；坚持稳中求进工作总基调，坚持新发展理念，坚持推进高质量发展，坚持以供给侧结构性改革为主线，坚持深化市场化改革、扩大高水平开放，加快建设现代化经济体系；统筹推进稳增长、促改革、调结构、惠民生、防风险各项工作，进一步稳就业、稳金融、稳外贸、稳外资、稳投资、稳预期，保持经济运行在合理区间，着力激发微观主体活力，提振市场信心，提高人民群众获得感、幸福感、安全感；守好发展和生态两条底线，继续打好三大攻坚战，推进三大战略行动，紧扣“一品一业、百业富贵”发展愿景，坚持扩大开放兴市、振兴实体强市、广聚人才旺市，持续做大城市规模、做强实体经济、做美生态环境、做好民生保障，围绕把贵阳打造成为在西部地区有影响力的国际化都市、中高端消费品贸易之城和制造之城的目标，苦干实干、拼搏奋斗，为全面建成小康社会收官打下决定性基础，以优异成绩迎接中华人民共和国成立70周年。

　　今年经济社会发展主要预期目标是：地区生产总值增长10%左右，规模以上工业增加值增长8%以上，固定资产投资增长13%，社会消费品零售总额增长10%，进出口总额增长15%，绿色经济、新经济增加值占地区生产总值比重分别达到45%、23%，一般公共预算收入增长6%，城乡居民人均可支配收入分别增长9%、9.5%，城镇登记失业率控制在4.2%以内，居民消费价格涨幅控制在省调控目标范围内，森林覆盖率达到53%，PM2.5浓度控制在每立方米35微克以内，新增能源消费总量控制在74万吨标准煤以内，完成省下达的节能减排降碳目标。

　　确保上述目标的实现，今年的政府工作要在聚力抓好十项重点工作上取得实效。

　　（一）在强合作、引资源上聚力，推动高水平对外开放取得实效。以高水平对外开放推动高质量发展，我们必须在历史前进的逻辑中前进，在时代发展的潮流中发展。

　　积极融入“一带一路”建设、长江经济带发展、粤港澳大湾区建设等国家战略，以中高端消费和中高端制造为引擎，坚持“市场引领、贸易先行、以贸促工、工贸并进”的原则，加快建设国家内陆开放型经济试验区核心区，形成具有贵阳特色的高水平对外开放新格局。强化互联互通。全力推进改貌铁路口岸建设，加快申报木材、粮食等进口指定口岸；积极推动中欧班列、国际陆海贸易新通道班列常态化运行，新开通6条国际航线；加快推进国际邮件互换局、国际快件中心和保税物流中心建设，着力打造国际陆海贸易新通道物流节点。强化平台支撑。进一步优化完善开发区管理体制机制，加大政策支持和资源配置力度，有序推进开发区空间拓展；以大数据创新发展为抓手，加快把贵阳国家高新技术产业开发区培育成为引领贵阳高质量发展的强大引擎；依托军工资源优势，加快把贵阳经济技术开发区培育成为国家军民结合（装备制造）高新技术产业化基地；发挥好功能作用和政策优势，加快把贵阳综合保税区培育成为贵州内陆开放型经济试验区的桥头堡；利用好临空经济优势，加快把贵州双龙航空港经济区培育成为富有特色的高质量国家级临空经济示范区；规划建设中欧产业园，大力推进瑞士（贵州）产业示范园、中英产业园等国际合作园区建设。强化交流合作。筹办好2019中国国际大数据产业博览会、生态文明贵阳国际论坛等活动，加快构建“一带一路”经贸文化信息交流平台，不断扩大贵阳的影响力和美誉度。强化产业招商。深入推进产业大招商行动，加快构建全球招商引业平台，面向欧洲、日韩、东盟、南亚等地区和京津冀、长三角、珠三角、粤港澳大湾区等开展精准招商，全年新引进产业项目500个以上，实际到位资金1265亿元以上，增长15%以上，其中工业项目到位资金占30%以上；实际利用外资17.8亿美元，增长13%，进出口总额40亿美元，增长15%；围绕把国家级开发区打造成为国际化高端消费品制造核心区的目标，积极引进国际知名高端消费品制造企业和产品；围绕把云岩区、南明区、观山湖区打造成为国际化高端消费品工贸并进示范区的目标，积极引进国际知名卖场、高端消费品制造企业和产品；围绕把花溪区、乌当区、白云区打造成为中高端大众消费品制造核心承载区的目标，积极引进中高端大众消费品制造企业和产品、生产性服务业企业和平台；围绕把清镇市、修文县、息烽县、开阳县打造成为中高端制造业核心配套区的目标，积极引进中高端消费品制造配套企业和产品。强化配套服务。进一步完善市级口岸协调机制，提升贵阳口岸通关便利化水平；认真落实外商投资企业准入前国民待遇加负面清单管理制度，积极争取72小时过境免签政策；设立开放型经济发展专项资金，发挥好市级出口退税资金池作用。强化人才引进。设立境外招才引智工作站，实施重点产业领军人才工程，谋划一批具有特色风情的商业街区、人才小镇，建设国际化社区、国际化医院和国际化学校，增强对人才的吸引力。

　　（二）在调结构、兴产业上聚力，推动实体经济做强取得实效。实体经济是贵阳发展的根基，是高水平对外开放推动高质量发展的重要抓手，我们必须下大决心、花大力气，全力以赴兴实业、强实体。

　　认真落实“巩固、增强、提升、畅通”八字方针，加快推动供给侧结构性改革，大力推进质量变革、效率变革、动力变革，推动产业结构迈向中高端，加快构建现代化经济体系。深入实施市场主体培育“四转”工程，新增规模以上工业企业100个、规模以上服务业企业100个、上市和新三板挂牌企业2个。大力发展中高端制造业。以打造中高端消费品制造基地为目标，统筹推进工业发展倍增计划和“双千工程”，改造提升四大传统支柱产业，培育发展五大战略性新兴产业，积极争创制造业高质量发展国家级示范区；以德国、日本、韩国等为代表的制造业强国为目标，新引进培育不少于10个中高端制造和商贸服务优强企业，推进吉利整车制造和发动机项目达产增效，推动宝能新能源汽车产业园、中电新能源汽车西南产业园加快建设，比亚迪贵阳智能制造产业基地开工建设，新引进15个以上汽车配套企业，汽车制造业产值比上年翻两番。加快发展中高端商贸业。以建设中国（贵阳）跨境电子商务综合试验区为抓手，推进跨境电子商务公共服务平台和线下体验店建设，建成西部进口商品展销中心；实施“百店引进”计划，引入各类中高端消费品商贸主体，布局建设中高端商业综合体，推动现有的商业综合体提质升级，打造高水平新型商圈，新增限额以上批发、零售、住宿、餐饮企业150个以上，塑造“爽爽的贵阳，购物的天堂”消费品牌。转型发展传统优势产业。全面完成中铝退城进园，力争引进培育5个规模以上铝精深加工项目，铝及铝加工业产值增长8%；加快推进开磷集团以渣定产、绿色转型，积极推进磷石膏新型建筑建材基地建设。培育发展战略性新兴产业。加快推进陶氏化学水性环保材料产业园、玖行汽车充换电产业及国家级新能源示范中心等新能源、新材料项目建设，汉能移动新能源项目一期实现量产、二期完成厂房建设和设备安装；建成德威智能制造等项目；推进国家西部医疗健康大数据中心及产业园、乌当区和修文县全省大健康医药产业示范区县建设，医药食品产业产值增长10%；积极开展国家军民融合创新示范区创建工作，大力推动军转民、民参军，实施军民融合项目20个以上。积极发展生产性服务业。大力实施“互联网+物流”行动计划，建成清镇国家电商物流园、贵州跨境电商产业园、改貌铁路物流中心，推进贵阳铁路枢纽都拉营货运场站专用线项目（一期）建设，引进生产性服务业企业50个以上，规模以上交通运输、仓储和邮政业营业收入增长10%。着力发展文化旅游产业。加快创建国家全域旅游示范区，大力推动天河潭景区创建国家级旅游度假区，加快建设恒大文化旅游城、华强文化旅游小镇等项目，新增4A级旅游景区2至3个，新设海外旅游文化营销推广中心5个，全年接待游客人次、实现旅游总收入分别增长20%、25%以上。

　　（三）在抓强化、促融合上聚力，推动数字经济发展取得实效。大数据是一个时代、一种潮流。我们必须乘势而上、顺势而为，把中国数谷的品牌做得更加响亮，为实现高质量发展提供强大动能。

　　坚持“四个强化”，做实“四个融合”，坚定不移推进大数据战略行动，做强做优数字经济，加快建设国家大数据（贵州）综合试验区核心区。把创新作为大数据发展的核心，引进大数据优强企业60个，新增规模以上大数据企业50个，培育大数据核心竞争力品牌3个，大数据企业主营业务收入增长20%以上，力争高新技术企业突破1000个，全社会研究与试验发展经费投入占地区生产总值比重达到2%，科技进步对经济增长的贡献率达到63%。全力构建大数据产业体系。加快推进公安部大数据中心、戴尔国际软件服务外包基地、华为智能制造及数据中心、浪潮大数据产业园、腾讯云启（贵阳）创新基地和西南区域交付中心等引领性项目建设。全力构建大数据应用体系。启动“数智贵阳”建设，推出大数据智慧治理应用15个、大数据智慧民生应用10个；建成国家工业互联网标识解析二级节点，加快建设“一品一业、百业富贵”大数据支撑平台、国家IPv6大数据应用创新中心、贵阳智能制造协同共享平台、“万企融合”服务与监测平台，数博大道建设取得实质性成果，着力打造永不落幕的数博会；实施工业企业智能化改造项目50个，规模以上工业企业上云比例达到80%以上。全力构建大数据标准体系。开展5项国家标准示范验证和5项地方标准制定，打造大数据技术标准创新公共服务平台，完善国家技术标准创新基地（贵州大数据）贵阳区域分基地功能。全力构建大数据法规体系。认真实施《贵阳市健康医疗大数据应用发展条例》等大数据法规和规章，积极做好大数据领域立法工作。全力构建大数据安全体系。加快推进国家级大数据及网络安全示范试点城市、国家大数据安全靶场（二期）、大数据安全产业示范区、工业信息安全制造业创新中心建设，办好2019贵阳大数据及网络安全精英对抗赛。

　　（四）在拓空间、强功能上聚力，推动宜居城市建设取得实效。坚持人民城市为人民，不断提升城市环境质量、人民生活质量、城市竞争力，建设和谐宜居、富有活力、特色鲜明的现代化城市。

　　抓重点、破难点、抢时点、做亮点，进一步优化城市功能，提升城市能级量级。拓展城市空间。编制完成《贵阳空间发展战略（2020—2035）》，开展国土空间规划编制工作，优化完善市域空间格局和产业空间格局；以“三县一市”撤县（市）设市辖区为契机，推进北部新城建设，规划建设中高端消费品制造特色产业小镇，进一步提高区域融合发展水平；推进贵阳与贵安新区同城化发展、贵阳与遵义、安顺、黔南联动发展，加快构建黔中城市群。优化城市路网。强力推进基础设施“六网会战”专项行动，以大交通构建城市发展大格局，产业大道、贵阳至长顺高速公路等重大项目开工建设，加快推进旅游公路环线建设，启动修文至开阳、修文至乌当城市道路规划建设，力争人民大道南段、延安东路延伸段、花溪大道改造提升工程建成通车，实施城市路网联通工程，打通“断头路”20公里以上；加快轨道交通2号线、3号线建设，贵阳至成都高速铁路、市域快铁西南环线贵阳段建成通车。完善城市功能。实施棚户区城中村改造4.8万户、660万平方米，三马片区改造取得新进展；建设地下综合管廊10公里，建成海绵城市项目10个、防空地下室5万平方米；开工建设贵阳奥林匹克体育中心（二期）、花溪水库至南郊水厂、花溪水库至中曹水厂输水工程等重大项目；优化城市公交、轨道交通接驳换乘体系，完善高铁贵阳北站和东站、金阳客车站配套路网，增加公交线路7条，新增公共停车位1万个，新建人行过街设施11个；加快城市直联网、物联网、5G网络和智慧广电网络建设，提升信息基础设施水平。加强城市管理。深入开展城市乱象整治行动、文明城市提升行动，启动22个占道夜市摊区退街入室工作，完成汽配市场专项整治工作；建设智慧市政大数据监管平台，实施公共设施管理提升工程，常态化开展“三车”整治和机动车违停治理。

　　（五）在补短板、强弱项上聚力，推动乡村振兴取得实效。把乡村振兴战略作为新时代做好“三农”工作的总抓手，因地制宜、步步为营、扎实推进。

　　建立健全城乡融合发展体制机制和政策体系，着力推进“五个振兴”，真正让农业强起来、农村美起来、农民富起来。坚决打好脱贫攻坚战。以脱贫攻坚统揽经济社会发展全局，深入推进大扶贫战略行动，聚焦“两不愁三保障”目标，打好脱贫攻坚“2019春季攻势”，常态化开展扶贫领域突出问题“五个专项治理”，精准脱贫13200人以上，巩固提升64个贫困村脱贫成果；全面完成3019户、12090人易地扶贫搬迁任务，着力构建后续扶持“五个体系”，稳步推进搬迁群众市民化，确保有劳动力的搬迁家庭实现至少1人稳定就业、每个安置点建设1个卫生服务机构、搬迁群众子女就学需求得到满足、不因搬迁出现各类社会保障漏保断保；精准落实教育医疗住房“三保障”政策，确保不让一个孩子因家庭经济困难而失学、贫困人口看得起病、符合条件的农村住房应搬尽搬、应保尽保、应改尽改；打好农村饮水安全问题攻坚决战，解决11.96万农村人口饮水安全巩固提升问题，确保饮水不愁；加大脱贫攻坚对口帮扶力度，深入推进贵阳大市场带动全省大扶贫，实施产销对接、保供稳价体系建设工程，启动“互联网+”农产品进城工程，带动10万以上省内贫困人口实现脱贫。纵深推进农村产业革命。深入实施绿色农产品泉涌工程，农业产业结构调整20万亩，全面完成100万亩产业结构调整任务，粮经作物比重降低到1﹕9；新增省级乡村旅游点10个，打造精品乡村旅游线路5条，乡村旅游总收入180亿元；推动农业优势产业园区化、品牌化发展，加快建设开阳县富硒农产品加工示范区，新增省级现代高效农业示范园区3个，扶持农产品加工企业20家以上，农产品加工总产值850亿元以上、转化率52%以上。深入实施农村人居环境提升行动。进一步提高政治站位，深入开展“大棚房”问题专项清理整治，在100个村寨开展千村整治提升行动，建成特色小城镇6个、富美乡村示范村35个，县级以上文明村寨创建率100%。全面深化农村改革。实现农村“三变”改革行政村全覆盖，完成农村集体产权制度改革整市试点工作,实现村社合一、集体经济股份合作社全覆盖，确保所有贫困户都参加合作社，每个贫困家庭都能获得稳定的收入；积极开展城乡要素双向流动试点工作，建成农村产权综合交易中心4个；有序引导非农产业和农村人口向城市和小城镇转移，常住人口城镇化率提高到76%。

　　（六）在严保护、优环境上聚力，推动生态文明建设取得实效。发展经济绝不能以牺牲生态环境为代价，必须正确处理经济发展和生态环境保护的关系，推动形成人与自然和谐发展新格局。

　　坚持生态优先、绿色发展，坚决落实长江经济带“共抓大保护、不搞大开发”的要求，打好污染防治攻坚战，巩固中央环保督察反馈问题整改成效，大力推进生态文明制度创新，加快建设全国生态文明示范城市。打好碧水保卫战。实施城镇污水处理提质增效三年行动，建成再生水厂9个、日新增污水处理能力40.5万吨，提标改造再生水厂8个，巩固提升南明河治理成效；制定实施污水收集管网建设规划，完善雨污分流系统，城市生活污水处理率达到95%以上，集中式饮用水源地水质100%稳定达标，全面消除黑臭水体。打好蓝天保卫战。突出抓好城市扬尘、工业废气、燃煤管控、柴油车尾气、夜市油烟等污染源治理，持续开展绿美贵阳行动计划，完成植树造林及新造林抚育12万亩、山体治理2000亩，新增城市绿地100万平方米，环境空气质量优良率保持在95%以上。打好净土保卫战。实施农业环境突出问题治理工程、农业废弃物资源化利用整县推进工程和化肥农药使用零增长计划，开展重点行业企业用地土壤污染状况详查，实行建设用地准入环境管理，严控污染地块再开发利用。打好固废治理战役。落实好固废工程性治理措施，工业固体废物安全处置率达到98%以上，开磷集团交椅山磷石膏渣场治理取得明显成效；加快推进城市生活垃圾焚烧发电项目和餐厨废弃物处置项目建设，生活垃圾分类处理覆盖率达到60%以上；积极发展节材环保装配式建筑，装配式建筑在新建住宅中使用比例达到15%。打好乡村环境整治战役。大力开展农村厕所革命、农村生活垃圾治理、农村生活污水治理、村庄清洁专项行动，新建、改造农村村级公共卫生厕所325个、农村户用卫生厕所9000户，大型养殖场实现粪污处理设施装备全配套，力争90%左右的村庄生活垃圾得到有效治理，农村生活污水治理能力明显提高。

　　（七）在优服务、增活力上聚力，推动重点领域改革取得实效。贵阳经济社会的加速发展、跨越发展，只有依靠改革这个历史前进的动力前进、顺应改革这个时代发展的潮流发展，才能不断争创贵阳发展的新优势。

　　以打造政务服务最优城市为目标，加快建立公平开放透明的市场规则和法治化营商环境。深化“放管服”改革。全面落实“非禁即入”要求，严格执行市场准入负面清单，加强社会信用体系建设，建成贵阳市优化营商环境大数据平台，持续开展“减证便民”行动，企业开办审批通过“一网通办”系统办理1个工作日以内完成，工程建设项目审批80个工作日以内完成，政务服务事项网上办理率达到100%，100个高频事项的办理最多跑一次。深化财税体制改革。全面实施零基预算管理和预算绩效管理，全面落实国家减税降费政策，对小微企业和科技型初创企业实施普惠性税收减免，继续执行阶段性降低失业和工伤保险费率政策，全面实施修改后的个人所得税法，切实减轻个人税负。深化投融资体制改革。建立政府投资正面清单管理制度，探索推行企业投资项目建设承诺制，积极鼓励社会资本进入提供公共产品和公共服务的领域。深化国有企业改革。完成市属国有企业战略性重组工作，建立健全国有企业法人治理结构，积极开展混合所有制改革，优化整合资源资产配置，降低企业资产负债率，真正让企业实现实业做强、主业做优。支持民营经济加快发展。抓紧制定出台贵阳市支持民营经济发展的政策措施，加快民营经济服务平台建设，深入开展拖欠民营企业资金问题专项治理，设立民营企业纾困基金和小微企业政策性融资担保基金，扶持微型企业3000家以上，民营经济增加值占地区生产总值比重达到58%。

　　（八）在稳投资、扩内需上聚力，推动重大项目建设取得实效。在高质量发展中，新的投资领域层出不穷，企业投资活力全面释放，我们要坚定发展信心，保持战略主动，以有效投资稳住经济发展基本盘，保持稳中求进的良好态势。

　　坚持抓发展必须抓投资，不断做大投资增量、壮大经济总量、增强发展后劲。抓实项目储备。进一步完善和充实投资项目三年滚动计划，确保市级重大项目库规模动态保持在3万亿元以上。抓好前期工作。积极争取国家和省前期工作资金支持，抓好水资源保障战略研究和优化配置、区域融合道路联网工程等一批战略性、基础性重大项目前期工作。抓紧项目建设。新开工建设项目824个，年度投资1500亿元，完成农业农村投资43亿元、交通基础设施投资127亿元、教育投资45亿元、水务投资18亿元，抓好工业项目集中开工、集中投产活动，确保新开工建设工业项目60个、建成投产70个，完成工业投资260亿元。抓足项目融资。抢抓国家较大幅度增加地方政府专项债券规模等机遇，强化融资服务平台建设，规范有序推进政府和社会资本合作，发挥好资本市场和非银行金融机构融资功能，争取更多的资金支持；按照不低于5%的比例压减一般性财政支出，增加对脱贫攻坚、“三农”、结构调整、科技创新、生态环境、民生等领域的投入。抓严项目管理。进一步加强投资管理和项目督查，建立“政府定标准，企业作承诺，过程强监管，失信重惩戒”的企业投资项目建设管理新模式；建立重点项目专班服务机制，营造良好建设环境。

　　（九）在办实事、促共享上聚力，推动民生改善取得实效。保障和改善民生没有终点，只有连续不断的新起点；民生改善不仅仅是发展的目的，更是让发展迈向高质量的动力。

　　在高质量发展中，我们要坚定不移增进民生福祉，不断满足人民对美好生活的向往，把民之所盼作为政之所向，把民之所需作为政之所为，把民生实事办好、办实，让发展指标的幸福含量更高、发展成果的民生比重更大。促进充分就业。深入实施高校毕业生就业创业促进计划和基层成长计划，扎实开展农民全员培训三年行动计划，全年新增城镇就业18.5万人以上，农村劳动力转移就业3.76万人，动态消除城乡“零就业家庭”，加快创建充分就业城市。保持物价稳定。建设城市保供基地1万亩、社区智慧微菜场50个，建成贵阳市肉类交易中心批发市场，进一步降低生鲜农产品价格。优先发展教育。加快推进“百校攻坚”行动计划和“全面改薄”工作，新建、改扩建中小学、幼儿园181所，新增学位3万个以上，学前教育普惠率达到80%；开展优质示范高中创建，推动清镇职教城内涵式发展，夯实中职基础，推进高校“双一流”学科建设，确保教师平均工资收入水平不低于当地公务员平均工资收入水平。建设健康贵阳。深入开展健康城市建设试点工作，进一步调整医疗服务价格和降低公立医院药占比，完成15个社区卫生服务中心标准化建设，建成30分钟乡村健康服务圈，深入开展“互联网+医疗健康”便民惠民活动，居民实名就诊率达到80%以上；扎实抓好食品药品安全监管，争创国家食品安全示范城市。繁荣文体事业。市少年儿童图书馆主体工程完成,建设社区图书共享系统50个,开展“全民健身贵阳行”系列活动，持续办好贵阳国际马拉松赛等重大活动和赛事，争创全民运动健身模范城市。健全保障体系。不断扩大社会保险覆盖面，健全完善城乡社会救助体系和低保标准与物价上涨挂钩联动机制；完善住房市场体系和住房保障体系，稳定地价房价，启动1万套安居房工程建设，推进公租房、保障房配套设施建设；完善养老护理体系，建成15个城乡社区日间照料中心、15个农村互助幸福院，农村生活不能自理特困人员集中供养率提高到45%。

　　（十）在防风险、促和谐上聚力，推动社会治理取得实效。树牢风险防范意识，保持社会大局和谐稳定，为人民群众筑起牢不可破的安全网。

　　持续打好防范化解重大风险攻坚战，构建新时代共建共治共享的社会治理新格局。防范金融风险。严格落实政府性债务管理“七严禁”和“1+8”系列政策，开展全市政府隐性债务再核查和政府债务风险突出地区专项督查，采取有效措施，减少政府债务存量、严控政府债务增量，坚持净地出让和低效用地有效利用“两条腿”走路，实施好土地储备和土地出让“两个计划”，建好资金池和土地池“两个池子”，保证发展资金需要；完善贵阳大数据防范金融风险系统平台，规范整治互联网金融，严厉打击非法集资，守好不发生区域性系统性金融风险的底线。防范社会风险。健全重大决策社会稳定风险评估机制，提高重大决策群众参与度；完善矛盾纠纷多元调解机制，加大解决历史遗留问题力度；严厉打击传销违法犯罪行为，大力实施社会治理智慧提升工程，加快推进“平安贵阳”建设；深入开展“两严一降”、扫黑除恶专项斗争、禁毒人民战争，创建全国禁毒示范城市；严格落实安全生产责任制，全面加强自然灾害预测预防，坚决防止发生重大及以上生产安全事故，全力守护人民群众生命财产安全，努力把贵阳建设成为全国最安全的城市之一。

　　各位代表，新时代是大发展、大作为的时代。我们要以永不懈怠的精神状态和一往无前的奋斗姿态，铸就贵阳发展的新辉煌，让贵阳人民在建设公平共享创新型中心城市中共享幸福和荣光。

　　三、持之以恒抓好政府自身建设

　　我们的政府是人民政府。我们要牢记嘱托、感恩奋进，永远把人民放在心中最高位置，全心全意为人民服务，为人民利益和幸福勤奋工作，建设新时代人民满意的服务型政府。

　　（一）加强政治建设，始终做到忠诚为政。提高政治站位、锤炼政治能力，旗帜鲜明讲政治。认真开展“不忘初心、牢记使命”专题教育，增强“四个意识”，坚定“四个自信”，做到“两个维护”，坚定对马克思主义的信仰、对中国特色社会主义的信念、对实现中华民族伟大复兴中国梦的信心，坚持党的基本理论、基本路线、基本方略不动摇，始终在政治立场、政治方向、政治原则、政治道路上同以习近平同志为核心的党中央保持高度一致，严守政治纪律和政治规矩，坚决贯彻落实好中央、省委和市委的决策部署，以实干实绩诠释对党和人民的绝对忠诚。

　　（二）坚持厉行法治，始终做到依法行政。全面完成市级和区（市、县）机构改革，形成职责明确、依法行政的政府治理体系，增强政府公信力和执行力。恪守宪法法律，用法治思维和法治方式履行职责，严格规范公正文明执法，崇尚诚信、守信践诺。全面推进政务公开工作，养成在监督下履职的习惯，依法接受市人大及其常委会的法律和工作监督，自觉接受市政协的民主监督，全面接受监察监督，主动接受社会和舆论监督，让政府工作在法治轨道上稳步前行。

　　（三）永葆公仆本色，始终做到为民执政。牢固树立以人民为中心的发展思想，始终把人民对美好生活的向往作为我们的奋斗目标。政府要过紧日子，百姓要过好日子，加大民生投入，办好民生实事，民生支出占一般公共预算支出比例不低于65%。整合全市公共服务热线，实现“12345”一号集中受理，为企业和群众提供优质高效的服务。坚持干字当头、勤勉敬业，善始善终、善作善成，以踏石留印、抓铁有痕的劲头抓紧每一天、干好每件事，全心全意为贵阳人民谋幸福，为贵阳大地添光彩。

　　（四）勇于担当作为，始终做到高效施政。深入践行“五步工作法”，精准把握“八个关键细节”，不断提升研究问题、破解难题的能力。全面落实好中央、省委、市委激励广大干部新时代新担当新作为的措施，不断激发广大干部干事创业、比学赶超的内生动力。进一步规范督查考核工作，为基层减负松绑，激发干部崇尚实干、攻坚克难的责任担当。

　　（五）狠抓具体深入，始终做到实干勤政。坚持把抓具体、抓深入作为推动工作的认识论和方法论，坚决摒弃形式主义、官僚主义，深入一线、深入基层，多到困难大、矛盾多的地方去，紧盯要害环节，搞好调查研究，摸清具体情况，找准问题关键，拿出务实管用的办法措施，形成一级抓一级，层层抓落实，一鼓作气、一抓到底的干事创业氛围，推动问题一个一个解决，让抓具体抓深入在全市政府系统蔚然成风。

　　（六）严格正风肃纪，始终做到干净从政。严格落实中央八项规定及其实施细则精神，驰而不息整治“四风”、转变作风。加强全市政府系统领导干部政德建设，做到明大德、守公德、严私德，正心明道，防微杜渐，着力构建亲清新型政商关系。始终保持惩治腐败高压态势，盯紧盯牢公共资金、公共资源、公共工程等重点领域廉政风险，严惩群众身边的不正之风和腐败问题，扎实推进廉洁政府建设。

　　各位代表，我们前进的道路上困顿和坎坷不会少，困难和麻烦都还在。历史只会眷顾坚定者、奋进者、搏击者，不会等待犹豫者、懈怠者、畏难者。春风虽凛冽，未来更浩荡，方此“船到中流浪更急、人到半山路更陡”之时，初心越久，芳华越长。我们要以更宽阔的视野、更长远的眼光来思考问题、破解难题，要以坚如磐石的信心、只争朝夕的劲头、坚忍不拔的毅力去战胜前进中难以想象的惊涛骇浪，一步一个脚印地创造无愧于人民、无愧于历史的辉煌业绩！

　　各位代表，中国特色社会主义进入新时代，摆在我们面前的使命更光荣、任务更艰巨、挑战更严峻、事业更伟大。我们既然选择远航，就不惧惊涛骇浪。红日初升，其道大光。今天，历史的接力棒交到了我们手中，我们要肩负起历史赋予的使命和责任，在新时代的接力跑中，始终保持想跑的动力、会跑的定力、快跑的能力、能跑的耐力，心无旁骛、聚精会神地跑好属于我们的这一棒。

　　让我们紧密团结在以习近平同志为核心的党中央周围，在中共贵阳市委的坚强领导下，以自我革命、自我完善的勇气，以逢山开路、遇水架桥的锐气，以只争朝夕、敢教日月换新天的豪气，奋力书写新时代爽爽贵阳发展的新篇章，以优异成绩迎接中华人民共和国成立70周年！
